# Longitudinal Associations between the Built Environment, Biological Aging and Cognition

**DOI:** 10.64898/2026.10.08.26365061

**Published:** 2026-10-11

**Authors:** Faye Sanders, Vilte Baltramonaityte, Marlene Staginnus, Rosa H. Mulder, Ryan L. Muetzel, Martine Vrijheid, Erin C. Dunn, Helen L. Fisher, Charlotte A.M. Cecil, Esther Walton

## Abstract

Features of urbanicity have been linked to poor psychiatric health in adulthood, but little is known about their relationship with biological age and cognition earlier in life.

Using the Avon Longitudinal Study of Parents and Children (ALSPAC), we tested associations between the built environment and epigenetic ages (EpiAge _Altum_ , EpiAge _Horvath_ , EpiAge _PACE_ ) (n=2,452), brain age (n=751), and cognition (n=12,199). Analyses were replicated in Generation R and triangulated in the Millennium Cohort Study.

The built environment was not associated with epigenetic ages (β _Altum_ range = 0.013-0.015, *p* range = .466-.689; β _Horvath_ range = -0.011-0.023, *p* range = .182-.728; β _PACE_ range = -0.045-0.013, *p* range = .131-.524) or brain age (β range = 0.022-0.028, *p* range = .337-.429). A negative association with cognition was observed in Generation R.

We provide no consistent evidence that childhood built environment exposure relates to biological age and cognition in adolescence, suggesting these relationships may emerge in later life.

## Full Text Availability

The license terms selected by the author(s) for this preprint version do not permit archiving in PMC. The full text is available from the preprint server.

